# Neonatal Azithromycin Exposure and Childhood Growth: Long-Term Follow-Up of a Randomized Controlled Trial

**DOI:** 10.4269/ajtmh.24-0016

**Published:** 2024-07-16

**Authors:** Mamadou Bountogo, Lucienne Ouermi, Clarisse Dah, Ali Sié, Boubacar Coulibaly, Alphonse Zakane, Thierry Ouedraogo, Mamadou Ouattara, Elodie Lebas, Ian Fetterman, Aimée Kimfuema, Thuy Doan, Thomas M. Lietman, Catherine E. Oldenburg

**Affiliations:** ^1^ Centre de Recherche en Sante de Nouna, Nouna, Burkina Faso;; ^2^ Francis I Proctor Foundation, University of California, San Francisco, California;; ^3^ Department of Ophthalmology, University of California, San Francisco, California;; ^4^ Department of Epidemiology & Biostatistics, University of California, San Francisco, California

## Abstract

Single-dose azithromycin is being considered by the WHO as an intervention for prevention of child mortality. However, concerns have emerged related to longer term unintended consequences of early life antibiotic use, particularly among infants. We conducted a long-term follow-up in a random sample of children who had been enrolled in a trial of neonatal azithromycin versus placebo for prevention of mortality to assess whether neonatal azithromycin exposure led to differences in child growth up to 4 years of age. We found no evidence of a difference in any anthropometric outcome among children who had received a single oral dose of azithromycin compared with placebo during the neonatal period. These results do not support long-term growth-promoting or deleterious effects of early life azithromycin exposure.

## INTRODUCTION

Exposure to antibiotics during early infancy has been hypothesized to affect immune system development, risk of obesity and atopic disease, and the microbiome later in childhood.^1^^,^^2^ Antibiotic exposure in the weeks after birth is thought to lead to long-term changes in the microbiome compared with later in infancy because the microbiome is immature during the neonatal period.^3^ As a result, there has been concern that neonatal antibiotic exposure may be more likely to lead to long-term changes in child health compared with antibiotic exposure later in childhood.

Single-dose azithromycin has been shown in high-mortality settings to prevent all-cause child mortality.^4^^,^^5^ One of the main concerns, however, has been the potential long-term impacts on children of early antibiotic exposure, including selection for antimicrobial resistance and risk of atopic disease and obesity.^6^ Previous studies have been unable to demonstrate an effect of azithromycin on weight gain up to 6 months after treatment administration.^7^^–^^9^ We conducted a long-term follow-up of children originally enrolled in a trial of neonatal azithromycin administration for prevention of infant mortality in Burkina Faso, a setting with high neonatal mortality and a high prevalence of undernutrition.^10^ We followed children up to 4 years of age to understand long-term effects of neonatal azithromycin treatment on anthropometric endpoints.

## MATERIALS AND METHODS

### Parent trial.

The *Nouveaux-nés et Azithromycine: une Innovation dans le Traitemant des Enfants* (NAITRE) trial was a 1:1 randomized, placebo-controlled trial designed to evaluate whether a single, oral dose of azithromycin (20 mg/kg) administered to neonates reduced all-cause infant mortality compared with matching placebo (clinicaltrials.gov NCT03682653).^11^ Complete methods for the trial have been previously reported.^11^^,^^12^ Neonates were eligible for the trial if they were between 8 and 27 days of age, were able to feed orally, and weighed at least 2,500 g at the time of enrollment. Participants were enrolled from April 2019 through December 2020. The original trial and the long-term follow-up were reviewed and approved by the Comité d’Ethique pour la Recherche en Santé (National Research Ethics Committee) in Ouagadougou, Burkina Faso and the Institutional Review Board (IRB) at the University of California, San Francisco. Written informed consent was obtained from the caregiver of each enrolled child for all study activities, including a separate consent process for the long-term follow-up component of the study.

### Long-term follow-up recruitment.

A random sample of children were enrolled in long-term follow-up from June until September 2023 (between 30 and 49 months of age). Children were recruited from Nouna, Banfora, and Do districts in Burkina Faso. A list of randomly selected children was generated for inclusion in the long-term follow-up. An attempt to reach each caregiver was made three times by telephone and different times of day (e.g., morning, mid-day, and afternoon). If a given child could not be found, the study team moved to the next child on the list until the enrollment target (*N* = 2,250, with a maximum IRB-approved enrollment of 2,500) was reached. Study procedures occurred at a health facility. If a child was sick at the time of the long-term follow-up, they were referred to care at the facility. The target sample size for the long-term follow-up was based on comparison of Simpson’s alpha diversity in the gut microbiome by arm in subgroups of children defined by birthweight, age at enrollment, rural versus urban dwelling, and season. A sample size of 100 children per subgroup per arm was estimated to yield a difference of 1.7 units in Simpson’s diversity, allowing for 25% loss to follow-up.

### Intervention.

Neonates were randomized in a 1:1 fashion to a single, oral, directly observed dose of azithromycin (20 mg/kg) or equivalent volume of matching placebo at enrollment. The original study team was unmasked after the primary endpoint, whereas data collectors for the long-term follow-up were not aware of the child’s randomized treatment assignment.

### Anthropometric measurements.

We extracted the most recent height, weight, and mid-upper arm circumference (MUAC) measurements for each child from government-issued vaccination cards. These cards serve as a medical record for early childhood, and during each health care encounter, these measurements are entered in a standardized form in the health card. Primary healthcare facilities in the study area use standard UNICEF wooden height boards and scales for measuring height and weight. We extracted the most recent measurement for each child along with the date of the measurement and calculated the age of the child at the time of the measurement based on the child’s date of birth. Because anthropometric measurements were the most recent recorded in a child’s health card, the child’s age at the time of anthropometric measurement did not necessarily correlate with age at follow-up. Children whose most recent anthropometric measurements were at <12 months of age were excluded from the analysis. Baseline anthropometric measurements were collected at enrollment prior to randomization and treatment as previously reported.^7^

## STATISTICAL ANALYSES

Weight-for-age (WAZ), weight-for length (WLZ) or weight-for-height (WHZ), and length-for-age Z-scores (LAZ) or height-for-age Z-scores (HAZ) were calculated using 2006 WHO child growth standards^13^; WLZ and LAZ were calculated for children <24 months and HAZ and WHZ for children ≥24 months. Z-score values considered to be outliers per WHO child growth standards for WAZ (–6 to +5 SD), LAZ/HAZ (–6 to +6 SD), and WLZ/WHZ (–5 to +5 SD) were not included in the analyses. We excluded WHZ scores for children whose weight and height measurements were not collected during the same month from analyses. Because child growth trajectories change with age, we stratified analyses by age at anthropometric measurement: 12–23 months, 24–35 months, and 36–47 months. A linear regression model was used for each outcome, with the child’s randomized treatment assignment (azithromycin or placebo), the baseline value of each anthropometric measurement, and the child’s age at the follow-up visit (in months) as covariates. As a sensitivity analysis, we included all children regardless of age at anthropometric measurement in a model adjusting for the baseline value of the outcome and the child’s age at follow-up. A second sensitivity analysis stratified results by the child’s sex. All analyses were intention-to-treat and were conducted in Stata version 17.0 (StataCorp, College Station, TX).

## RESULTS

**Figure 1.**
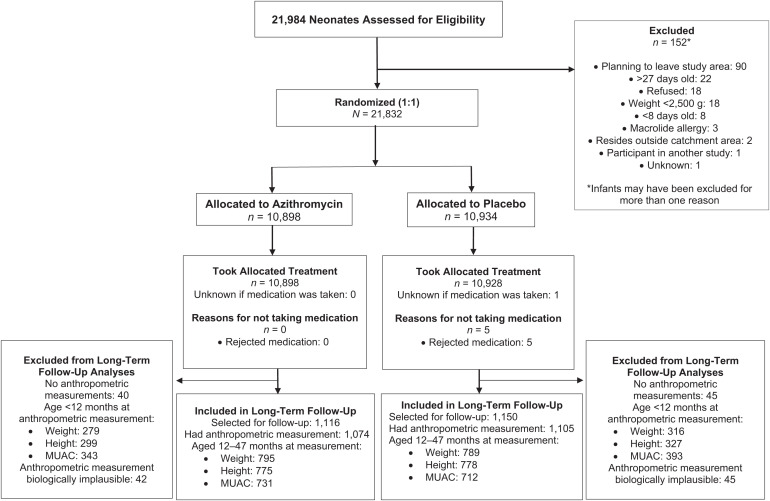
Screening, randomization, and follow-up of participants. MUAC = mid-upper arm circumference.

Of 21,832 neonates enrolled in the parent trial, 2,266 were enrolled in the long-term follow-up sample and 2,181 (96%) had anthropometric measurements available in their health card. Of these, 1,554 (71%), 1,585 (73%), and 1,439 (66%) had height, weight, and MUAC measurements between 12 and 48 months of age and were included in the analysis (Figure 1). Enrollment characteristics were similar among children included and not included in the long-term follow-up and by randomization arm, although children in the long-term follow-up more often lived in an urban setting and more often had delayed initiation of breastfeeding (Table 1). Median age at follow-up and anthropometric measurement was similar between the two arms (Table 2).

**Table 1:** Baseline characteristics* among neonates who were (*N* = 2,266) and were not (*N* = 19,568) enrolled in the NAITRE long-term follow-up

Characteristic	Enrolled in Long-Term Follow-Up		Not Enrolled in Long-Term Follow-Up	
	Azithromycin	Placebo	Azithromycin	Placebo
*N*	1,116	1,150	9,784	9,784
Age of Enrollment, days, median (IQR)	10 (9–14)	10 (8–14)	11 (9–15)	11 (9–14)
Sex				
Female	549 (49.3%)	564 (49.0%)	4,864 (49.7%)	4,867 (49.7%)
Male	565 (50.7%)	586 (51.0%)	4,920 (50.3%)	4,917 (50.3%)
Urban Dwelling				
Urban	1,037 (93.1%)	1,082 (94.1%)	7,923 (81.0%)	7,973 (81.5%)
Rural	77 (6.9%)	68 (5.9%)	1,855 (19.0%)	1,801 (18.4%)
Missing	0 (0.0%)	0 (0.0%)	6 (0.06%)	10 (0.1%)
Birthweight, g				
Median (IQR)	3,000 (2,700–3,300)	3,000 (2,700–3,300)	3,000 (2,700–3,229)	3,000 (2,700–3,250)
Weight at Enrollment, g				
Median (IQR)	3,325 (3,001–3,658)	3,305 (2,953–3,659)	3,300 (2,980–3,620)	3,295 (2,995–3,610)
Length at Enrollment, cm				
Median (IQR)	50.5 (49.3–52.0)	50.5 (49.2–52.0)	50.4 (49.3–51.9)	50.5 (49.4–52.0)
WLZ, mean (SD)	−0.56 (1.29)	−0.56 (1.28)	−0.63 (1.31)	−0.66 (1.29)
WAZ, mean (SD)	−0.56 (0.97)	−0.56 (0.10)	−0.63 (0.93)	−0.61 (0.93)
LAZ, mean (SD)	−0.52 (1.10)	−0.51 (1.12)	−0.54 (1.04)	−0.50 (1.06)
MUAC, median (IQR)	10.5 (10.0–11.5)	10.5 (10.0–11.5)	11.0 (10.0–11.5)	11.0 (10.1–11.5)
Mother’s Age, median (IQR)	26 (21–31)	26 (21–31)	25 (21–30)	25 (21–30)
Mother’s Education				
None	660 (59.3%)	651 (56.6%)	5,250 (53.7%)	5,378 (55.0%)
Primary	174 (15.6%)	194 (16.9%)	1,816 (18.6%)	1,784 (18.2%)
Secondary or Higher	280 (25.1%)	305 (26.5%)	2,717 (27.8%)	2,618 (26.8%)
Missing	0 (0.0%)	0 (0.0%)	1 (0.01%)	4 (0.04%)
No. Children in Household, median (IQR)	2 (1–3)	2 (0–3)	1 (0–3)	1 (0–3)
Pregnancy Type				
Singleton	1,086 (97.5%)	1,128 (98.1%)	9,616 (98.3%)	9,625 (98.4%)
Multiple	28 (2.5%)	22 (1.9%)	167 (1.7%)	155 (1.6%)
Missing	0 (0.0%)	0 (0.0%)	1 (0.01%)	4 (0.04%)
No. of Antenatal Visits, median (IQR)	4 (3–5)	4 (3–5)	4 (3–5)	4 (3–5)
Initiation of Breastfeeding				
Immediate	1,022 (91.7%)	1,026 (89.2%)	9,298 (95.0%)	9,315 (95.2%)
Delayed	89 (8.0%)	119 (10.3%)	477 (4.9%)	455 (4.7%)
Not Breastfeeding	3 (0.3%)	5 (0.4%)	8 (0.08%)	10 (0.1%)
Missing	0 (0.0%)	0 (0.0%)	1 (0.01%)	4 (0.04%)

IQR = interquartile range; LAZ = length-for-age Z-score; MUAC = mid-upper arm circumference; WAZ = weight-for-age Z-score; WLZ = weight-for-length Z-score.

* Baseline characteristics were collected via caregiver survey and clinical exam during the baseline assessment of the parent trial.

**Table 2:** Anthropometric outcomes stratified by age at follow-up by randomized treatment assignment among children aged 12 to 47 months at the time of anthropometric measurement*

Characteristic	*N*	Azithromycin	Placebo	Mean Difference^†^ (95% CI)	*P*-Value
Age at Follow-up, months, median (IQR)	–	39 (36–43)	39 (36–43)	–	–
Age at Anthropometric Measurement, months, median (IQR)					
Weight Measurement	–	33 (15–40)	32 (15–39)	–	–
Height Measurement	–	33 (15–40)	27.5 (15–39)	–	–
MUAC Measurement	–	33 (16–40)	33 (15–40)	–	–
Weight, kg, mean (SD)					
12–23 months	715	9.4 (1.1)	9.5 (1.1)	−0.07 (–0.22 to 0.08)	0.37
24–35 months	196	12.4 (1.8)	12.4 (2.2)	−0.16 (–0.67 to 0.34)	0.53
36–47 months	674	13.5 (1.7)	13.6 (1.8)	−0.13 (–0.38 to 0.12)	0.31
Height, cm, mean (SD)					
12–23 months	710	76.2 (3.9)	76.2 (3.7)	0.04 (–0.42 to 0.50)	0.87
24–35 months	195	88.1 (6.1)	87.4 (7.2)	0.03 (–1.71 to 1.76)	0.98
36–47 months	649	92.3 (6.1)	92.2 (6.8)	0.01 (–0.95 to 0.97)	0.99
WAZ, mean (SD)					
12–23 months	715	−0.63 (1.0)	−0.54 (1.0)	−0.03 (–0.16 to 0.10)	0.62
24–35 months	196	−0.72 (1.1)	−0.79 (1.4)	−0.05 (–0.40 to 0.29)	0.76
36–47 months	674	−0.91 (0.95)	−0.84 (1.1)	−0.07 (–0.22 to 0.08)	0.37
WLZ/WHZ,^‡^ mean (SD)					
12–23 months	645	−0.22 (1.1)	−0.15 (1.1)	−0.05 (–0.22 to 0.11)	0.50
24–35 months	184	0.006 (1.2)	0.19 (1.7)	−0.26 (–0.67 to 0.16)	0.22
36–47 months	640	0.21 (1.3)	0.32 (1.2)	−0.12 (–0.31 to 0.08)	0.24
LAZ/HAZ, mean (SD)					
12–23 months	710	−0.94 (1.3)	−0.93 (1.3)	0.03 (–0.14 to 0.21)	0.70
24–35 months	195	−1.55 (1.6)	−1.67 (1.9)	0.02 (–0.48 to 0.52)	0.94
36–47 months	649	−1.80 (1.5)	−1.78 (1.7)	−0.005 (–0.25 to 0.24)	0.97
MUAC, cm, mean (SD)					
12–23 months	605	13.6 (1.5)	13.7 (1.5)	−0.11 (–0.34 to 0.13)	0.37
24–35 months	184	14.5 (1.2)	14.4 (1.3)	−0.04 (–0.40 to 0.33)	0.85
36–47 months	650	14.7 (1.2)	14.7 (1.2)	−0.03 (–0.22 to 0.16)	0.77

IQR = interquartile range; HAZ = height-for-age Z-score; LAZ = length-for-age Z-score; MUAC = mid-upper arm circumference; WAZ = weight-for-age Z-score; WHZ = weight-for-height Z-score; WLZ = weight-for-length Z-score.

* Children received a single oral dose of azithromycin or placebo as neonates (aged 8–27 days).

^†^ Adjusting for baseline measure and the child’s age at the time of long-term follow-up in months.

^‡^ Restricted only to children whose weight and height measurements were taken in the same month of age.

We found no evidence of a difference in any anthropometric endpoints in any age group (Table 2), although the 24- to 35-month age group was small and confidence intervals were wide. Results were robust to inclusion of all children regardless of age at anthropometric measurement (Supplemental Table 1). We found no evidence of a difference in outcomes by the child’s sex (Supplemental Table 2).

## DISCUSSION

In this analysis of children who were followed up to 4 years of age, we found no evidence of a difference in anthropometric measures in children who had received a single dose of azithromycin or placebo as neonates. Previous analyses from this study showed no difference in anthropometric endpoints at 6 months of age.^7^ However, mouse models have suggested that early-life antibiotic exposure may lead to changes in metabolism that are not apparent until later in life.^14^ The current results do not suggest that neonatal antibiotic exposure leads to long-term changes in anthropometric measurements in this population of children in Burkina Faso.

Strengths of this analysis include the many years of follow-up and the relatively large sample size. This analysis also has some limitations. The analysis relied on weight, height, and MUAC measurements that were collected routinely when children visited health facilities. The timing and accuracy of the measurements was therefore not standardized. We attempted to standardize timing by stratifying analyses by age at follow-up. Although there is likely measurement error due to use of nonstandardized measurements, extreme values were excluded from the analysis following WHO guidelines, and measurement error is unlikely to be differential by randomization arm. Bias introduced is likely to be toward the null. Approximately 25% of children enrolled in the longer term follow-up did not have anthropometric measurements recorded in their health card after 12 months of age. Children who did and did not have recent measurements may be substantively different, and this may have introduced selection bias into analyses. However, results were robust to inclusion of all children regardless of age at measurement. The trial did not collect data on maternal characteristics or gestational age that may be strongly predictive of child growth. However, because this was a randomized trial, we do not anticipate that adjustment for any baseline characteristics would affect inferences. We did not have data on why the child was seen at a health clinic (e.g., for a sick child visit or a routine check-up). Children visiting clinics for illness may have had lower anthropometric measurements than those attending well-child visits. For logistical and resource reasons, long-term follow-up was only conducted with approximately 10% of the original trial population, and thus some age-based subgroups were small. Due to small numbers, we were unable to assess differences in other subgroups of children, such as those based on baseline anthropometric measures.^15^ Larger studies with standardized follow-up times and measurements are needed to confirm these results.

In this long-term follow-up of children up to 4 years of age who previously received a single oral dose of azithromycin or placebo during the neonatal period, we were unable to demonstrate a difference in anthropometric measurements years after neonatal antibiotic exposure. Consistent with previous evidence suggesting no short-term effect of azithromycin on infant growth, we do not have evidence that single dose azithromycin during the neonatal period affects child growth trajectories later in life.

## Supplemental Materials

10.4269/ajtmh.24-0016 Supplemental Materials
