# Supplementary material for: Neonatal Azithromycin Exposure and Childhood Growth: Long-Term Follow-Up of a Randomized Controlled Trial
Source: Am J Trop Med Hyg. 2024 Jul 16;111(3):698–702. doi: 10.4269/ajtmh.24-0016 (PMC11376181; doi:10.4269/ajtmh.24-0016)
Supplement: Supplemental Materials [file tpmd240016.SD1.pdf]

Supplemental Table 1. Sensitivity analysis of long-term anthropometric endpoints by neonatal azithromycin or placebo including all children regardless of age at anthropometric measurement.

	N	Azithromycin	Placebo	Mean Difference[1] (95% CI)	P-value
Weight, kg	2,112	11.0 (2.5)	10.9 (1.5)	-0.05 (-0.16 to 0.07)	0.41
Height, cm	2,079	81.7 (10.2)	81.1 (10.2)	0.04 (-0.38 to 0.45)	0.87
WAZ	2,112	-0.48 (1.1)	-0.44 (1.2)	-0.01 (-0.10 to 0.08)	0.81
WHZ	1,959	0.13 (1.2)	0.17 (1.2)	-0.04 (-0.15 to 0.06)	0.42
HAZ	2,079	-1.0 (1.7)	-1.0 (1.8)	0.05 (-0.08 to 0.18)	0.44
MUAC	1,881	14.0 (1.5)	14.0 (1.5)	-0.04 (-0.16 to 0.08)	0.54

Abbreviations: CI, confidence interval; kg, kilograms; cm, centimeters; WAZ, weight-for-age Z-score; WHZ, weight-for-height Z-score; HAZ, height-for-age Z-score; MUAC, mid-upper arm circumference; [1]Adjusted for baseline measure of the outcome and the age at anthropometric measurement.

Supplemental Table 2. Long-term anthropometric endpoints by neonatal azithromycin or placebo stratified by child's sex. Models include all measurements regardless of child's age at anthropometric measurement, adjusted for the baseline measure of each outcome and the child's age at the time of outcome measurement.

	Female					Male				
	**N**	**Az**	**Placebo**	**Mean Difference[1] (95% CI)**	**P-value**	**N**	**Az**	**Placebo**	**Mean Difference[1] (95% CI)**	**P-value**
Weight, kg	1,037	10.9 (2.4)	10.9 (2.5)	-0.07 (-0.24 to 0.09)	0.38	1,075	11.1 (2.5)	11.0 (2.5)	-0.02 (-0.18 to 0.14)	0.78
Height, cm	1,023	81.3 (10.1)	81.3 (10.2)	-0.18 (-0.75 to 0.40)	0.55	1,056	82.0 (10.2)	80.9 (10.2)	0.25 (-0.33 to 0.84)	0.40
WAZ	1,037	-0.37 (1.1)	-0.32 (1.2)	-0.04 (-0.17 to 0.08)	0.50	1,075	-0.58 (1.2)	-0.57 (1.2)	0.009 (-0.12 to 0.14)	0.89
WHZ	963	0.31 (1.4)	0.33 (1.5)	-0.003 (-0.15 to 0.15)	0.97	996	0.14 (1.7)	0.30 (2.2)	-0.09 (-0.24 to 0.06)	0.25
HAZ	1,023	-0.95 (1.6)	-0.87 (1.7)	-0.06 (-0.23 to 0.12)	0.51	1,056	-1.1 (1.7)	-1.2 (1.8)	0.15 (-0.04 to 0.34)	0.12
MUAC	938	14.0 (1.5)	14.0 (1.5)	-0.07 (-0.24 to 0.11)	0.44	943	14.1 (1.5)	14.0 (1.5)	-0.008 (-0.18 to 0.16)	0.93

Abbreviations: CI, confidence interval; kg, kilograms; cm, centimeters; WAZ, weight-for-age Z-score; WHZ, weight-for-height Z-score; HAZ, height-for-age Z-score; MUAC, mid-upper arm circumference; [1]Adjusted for baseline measure of the outcome and the age at anthropometric measurement.
